# The impact of cognitive functioning on mortality and the development of functional disability in older adults with diabetes: the second longitudinal study on aging

**DOI:** 10.1186/1471-2318-6-8

**Published:** 2006-05-01

**Authors:** Lisa C McGuire, Earl S Ford, Umed A Ajani

**Affiliations:** 1 Division of Adult and Community Health, Centers for Disease Control and Prevention, 4770 Buford Highway, NE., MS K-66, Atlanta, GA 30328, USA

## Abstract

**Background:**

For older adults without diabetes, cognitive functioning has been implicated as a predictor of death and functional disability for older adults and those with mild to severe cognitive impairment. However, little is known about the relationship between cognition functioning on mortality and the development of functional disability in late life for persons with diabetes. We examined the relative contribution of cognitive functioning to mortality and functional disability over a 2-year period in a sample of nationally representative older US adults with diabetes who were free from cognitive impairment through secondary data analyses of the Second Longitudinal Study of Aging (LSOA II).

**Methods:**

Participants included 559 US adults (232 males and 327 females) ≥ 70 years old who had diabetes and who were free from cognitive impairment were examined using an adapted Telephone Interview of Cognitive Status (TICS), Activities of Daily Living (ADL), and Instrumental Activities of Daily Living (IADL).

**Results:**

Multivariate logistic regression was conducted to investigate the independent contribution of cognitive functioning to three mutually exclusive outcomes of death and two measures of functional disability status. The covariates included in the model were participants' sex, age, race, marital status, educational level, duration of diabetes, cardiovascular disease (CVD) status, and self-rated health. Persons with diabetes who had the lowest levels of cognitive functioning relative to the highest level of cognitive functioning had a greater odds of dying (AOR = 0.80, 95% CI = 0.67–0.96) or becoming disabled (AOR = 0.87, 95% CI = 0.78–0.97) compared to those people who were disability free.

**Conclusion:**

Older adults with diabetes and low normal levels of cognition, yet within normal ranges, were approximately 20% more likely to die and 13% more likely to become disabled than those with higher levels of cognitive functioning over a 2-year period. Brief screening measures of cognitive functioning could be used to identify older adults with diabetes who are at increased risk for mortality and functional disability, as well as those who may benefit from interventions to prevent or minimize further disablement and declines in cognitive functioning.

## Background

Cognitive dysfunction appears to be an additional complication of diabetes [1]. Accelerated declines in cognitive functioning have been consistently reported for older and middle-aged adults with diabetes [1-9]. In a recent systematic analysis, Cukierman and colleagues concluded that people with diabetes had a 1.2 to 1.5-fold greater change over time in measures of cognitive functioning and that the odds of future dementia was increased 1.6-fold[1]. In addition, the risk of cognitive decline was greater for those who had diabetes longer [4,5] and for those not treating their diabetes [4].

Older adults with diabetes have a heavy burden of disability and functional disability [8,10-14]. About 20–50% of people with diabetes report having a disability, which is 2–3 times more likely than the general population [13]. One-third of people with diabetes have difficulties with at least one activity of daily living (ADL, includes bathing, dressing, etc). In addition, diabetes is shown to be related to IADL limitations [15] as 57% have at least one limitation in instrumental activities of daily living (IADL, includes shopping, preparing meals, etc) [12].

Cognitive impairment and functional disability have similar trajectories associated age and are important public health issues of an aging population [15]. For older adults without diabetes, cognitive functioning has been implicated as a predictor of functional disability for older adults [3,15-21] and those with mild to severe cognitive impairment [22,23]. Diabetes is a common chronic condition in older adults that afflicts a large proportion of the aging population as approximately 10.3 million or 20.9% of adults aged ≥ 60 years old had diabetes in 2005 [24] and the diabetes prevalence rates for older adults have been increasing steadily since the 1980s [25]. However, little is known about the relationship between cognition functioning and the development of functional disability and mortality in late life for persons with diabetes. We examined the relative contribution of cognitive functioning to mortality and functional disability over a 2-year period in a sample of nationally representative older US adults with diabetes who were free from cognitive impairment through secondary data analyses of the Second Longitudinal Study of Aging (LSOA II) [26].

## Methods

### Participants

Data for the present investigation came from the LSOA II, a publicly available data set, which consists of a nationally representative sample with a multistage complex sampling design. The baseline of LSOA II was the 1994 National Health Interview Survey, Second Supplement on Aging II (SOA II), conducted by the Centers for Disease Control and Prevention after ethical approval for the study was obtained by the National Center for Health Statistics' Research Ethics Review Board. Participation in the survey was voluntary and confidentiality of respondents and their responses was assured by adherence to Section 308(d) of the Public Health Service Act (42 USC 242 m). In addition to receiving an advance letter describing the study prior to the interview, the interviewer described the voluntary nature of the study, participants' right to withdrawal at anytime, and the confidentiality measures employed. Informed consent then was obtained verbally from the participant when they gave their permission to proceed with the interview.

The SOA II was comprised of 9,447 community-dwelling, civilian men and women who were at least 70 years old (mean = 77.3, SE = .20). The follow-ups were conducted primarily using a Computer-Assisted Telephone Interview system. The data in the first follow-up, were collected between May 1997 and May 1998, while the second follow-up was obtained between June 1999 and August 2000. Cognitive measures were collected for the first time during the first follow-up; therefore, we limited our analyses to 559 individuals with diabetes who completed cognitive measures without proxy.

### Materials

Cognitive functioning was assessed at both follow-ups using an adapted telephone interview for cognitive status (TICS) [27]. Cognitive functioning assessed two independent factors of cognition: mental status (orientation, registration, and attention) and memory (immediate verbal memory). Participants' scores on the mental status items (0–10 points; e.g., Who is the president and vice-president?; What is used to cut paper?; What is a desert plant?; What is the day/date/month/year?; counting backwards from 20 and from 86) and memory task (0–10 points; 10 item list of concrete nouns) were summed resulting in a cognitive functioning score ranging from 0 to 20, with higher scores indicating higher levels of cognitive functioning. Methodologically, assessing cognitive functioning via the telephone is a reliable and valid approach [28-31]. Participants whose cognitive score was at least 1.5 standard deviation units below the mean performance were considered to have mild to severe cognitive impairment and were excluded from the analyses [32,33].

Diabetes was assessed by self-report, which is considered a reliable and valid indicator diabetes status [10,12,15,34-36]. Participants indicated whether they had diabetes at baseline and if they now have diabetes at first and second follow-ups. We considered people to have diabetes if they indicated "yes" to the diabetes questions.

Functional disability status was measured using self-reported ADLs and IADLs during the first and second follow-ups. ADLs, which are comprised of basic self-care tasks necessary for an adult to live independently, include bathing, dressing, eating, transferring, walking, and toileting, were assessed in the LSOA II. IADLs are higher-ordered skills that are also necessary for independent living. The IADL tasks assessed on the LSOA II were preparing meals, grocery shopping, managing money, using the telephone, doing light/heavy housework, and managing medications. Participants were classified into one of three mutually exclusive categories, death and two categories of functional disability status by their self-reported ability in the second follow-up to perform ADL and IADL tasks: 1). deceased – died between first and second follow-up as indicated by interviews with surviving family and friends; 2). ADL and/or IADL disabled – unable to perform at least one ADL task and/or at least one IADL task; and 3). disability free – able to perform all ADL and IADL activities. In addition, we controlled for participants functional disability status at the first follow-up.

Covariates modeled as predictors of mortality and functional disability status included measures of participants' sex, age, race, marital status, educational level, duration of diabetes, CVD, and self-rated health. Age, education, and duration of diabetes were continuous variables. The duration of diabetes was calculated based on self-reported year of diagnosis and year of first follow-up. Race was classified into two categories (white and nonwhite). Marital status was recoded into married or not married, with the not married classification including individuals who were widowed, never married, and separated/divorced. Cardiovascular disease (CVD) status was comprised of people who indicated that they had hypertension or that they had experienced a heart attack or a stroke. Self-rated health was recoded in two classifications (excellent or very good, good to poor).

### Statistical analyses

To account for the complex sampling design, SUDAAN statistical software, version 9.0 [37] was used in all statistical analyses [38]. Multivariate logistic regression models [39] were fit using the MULTILOG procedure with the GENLOGIT (generalized logit) option of SUDAAN after verifying the assumption of multicollinearity [40]. The multivariate logistic regression model was fit using the MULTILOG procedure of SUDAAN. Adjusted odds ratios (AOR) for each of the three levels of outcomes, mortality and functional disability status, and cognitive functioning relative to the disability free were provided along with their corresponding 95% confidence intervals.

## Results

Of the 9447 participants of the SOA II, 1183 died and 426 were missing in the 1^st ^follow-up, resulting in 7838 first follow-up participants. This sample of 7838 participants consisted of 1178 people with diabetes that was further reduced to 650 people with diabetes who completed the cognitive tasks at first follow-up without mild to severe cognitive impairment (*n *= 56); an additional 91 participants were excluded due to incomplete information for covariates, diabetes status, and outcome variables. There was no difference in age (*p *= 0.122), educational attainment (*p *= 0.799), and length of time with diabetes (*p *= 0.290) between those who were excluded due to incomplete information and those who were included in the sample. However, those who were excluded due to incomplete information compared to those who were included in the sample had lower first follow-up cognitive functioning (*p *= 0.003).

The analysis consisted of 559 older adults (232 males and 327 females) with diabetes and no evidence of mild to severe cognitive functioning. A first follow-up, they were on average 77.81 (SE = .17) years old and on average were diagnosed with diabetes 14.43 (SE = .43) years prior. The sample consisted of community-dwelling people who were primarily Caucasian (*n *= 477, weighted percentage 88.79%, *SE *= 1.39). More than half were female (*n *= 327, weighted percentage 58.00%, *SE *= 2.13), who were unmarried (*n *= 321, weighted percentage 56.28%, *SE *= 2.17), with more than 2 chronic condition (*n *= 307, weighted percentage 57.92%, *SE *= 2.02), with CVD (*n *= 399, weighted percentage 71.40%, *SE *= 2.14), who were disability free at first follow-up (*n *= 266, weighted percentage 48.64%, *SE *= 2.18) and rated their health as excellent or very good (*n *= 160, weighted percentage 28.14%, *SE *= 2.03) or as fair or poor (*n *= 399, weighted percentage 71.86%, *SE *= 2.03).

People with diabetes who rated their health as at least very good at first follow-up were more likely to be disability free than those people who rated their health as good or fair or poor (Table 1). People with diabetes who had more than 2 chronic health conditions were more likely to have died and to have become disabled, while those with fewer chronic conditions were disability free. Those with diabetes and CVD were more likely to have died or to have become disabled than those without CVD. People with diabetes without a functional disability tended to have higher levels of educational attainment and cognitive functioning than people who are dead or disabled. People with diabetes without a functional disability tended to have higher levels of educational attainment and cognitive functioning than people who died or became disabled. There was no relationship between death and functional disability status and duration of diabetes.

**Table 1:** Weighted Percentages or Means (Standard Error) of Mortality and Functional Disability Status among US Adults with Diabetes Mellitus aged ≥ 70, Longitudinal Study of Aging II, 1997/1998–1999/2000.

		Functional disability status at second follow-up*			
Characteristics	*n*	Dead (*n *= 67)	Disabled (*n *= 321)	Non-Disabled (*n *= 171)	*p-*value

Sex, %					0.06
Male	232	15.16 (2.30)	51.20 (3.15)	33.65 (3.17)	
Female	327	11.24 (1.84)	60.60 (2.48)	28.15 (2.18)	
					
Mean age at study entry, yrs	559	77.92 (0.51)^a^	78.18 (0.28)^b^	76.69 (0.27)^a,b^	<0.01
					
Race or Ethnicity, %					0.40
White	477	12.79 (1.45)	55.90 (2.16)	31.31 (2.07)	
Other	82	13.67 (5.03)	62.60 (6.89)	23.73 (5.02)	
					
Marital status, %					0.18
Married	238	13.64 (2.26)	52.04 (3.49)	34.32 (3.15)	
Unmarried	321	12.30 (1.92)	60.24 (2.54)	27.46 (2.41)	
					
Mean cognitive functioning	559	13.00 (0.30)^c^	13.41 (0.12)^d^	14.42 (0.18)^c,d^	<0.01
					
Mean educational attainment, yrs	559	10.75 (0.37)^e^	10.09 (0.20) ^f^	12.19 (0.22) ^e,f^	<0.01
					
Mean duration of diabetes, yrs	559	13.48 (0.97)	14.50 (0.62)	13.77 (0.85)	0.62
					
# Chronic conditions, %					<0.01
0–2	252	9.00 (1.92)	48.94 (3.47)	42.06 (3.52)	
>2	307	16.08 (2.18)	62.99 (2.58)	20.94 (2.26)	
					
CVD, %					<0.01
Yes	399	14.22 (1.74)	61.22 (2.43)	24.57 (2.10)	
No	160	9.57 (1.74)	45.25 (4.13)	45.17 (4.21)	
					
Prior Disability Status, %					<0.01
Disabled	293	14.98 (2.20)	72.56 (2.62)	12.45 (1.88)	
Non-Disabled	266	10.68 (1.93)	39.86 (3.19)	49.47 (3.21)	
					
Self-rated health, %					<0.01
Excellent/very good	160	7.06 (2.06)	42.57 (3.69)	50.37 (3.87)	
Good/fair/poor	399	15.17 (1.84)	62.17 (2.32)	22.67 (2.09)	

TOTAL	559	12.89 (1.40)	56.65 (1.98)	30.46 (1.84)	

Abbreviations: ADL, activities of daily living; IADL instrumental activities of daily living; NA not applicable.

Items with like subscripts are statistically significant from each other (p < 0.05).

* Weighted estimates.

A series of multivariate logistic regression models predicting the combined 2-year mortality and functional disability status were calculated after controlling for the covariates of participants' sex, age, race, marital status, educational level, duration of diabetes, CVD, and self-rated health. The multivariate logistic regression models allowed for multiple outcomes of mortality and functional disability to be compared simultaneously to the reference group of disability free. Persons with diabetes who had the lowest levels of cognitive functioning relative to the highest level of cognitive functioning had greater odds of dying (AOR = 0.80, 95% CI = 0.67–0.96) or becoming disabled (AOR = 0.87, 95% CI = 0.78–0.97). Furthermore, for death and functional disability, a graded relationship with cognitive functioning was apparent.

## Discussion

In this longitudinal study of US adults with diabetes aged ≥ 70 years who were functioning cognitively in the normal range, poor cognitive functioning was a significant and independent predictor of subsequent mortality and the development of functional disability. Persons with diabetes and lower levels of cognitive functioning were more likely to die or to become functionally disabled than those with the higher levels of cognition. Interestingly, length of diabetes was not related to mortality or functional disability.

Consistent with previous research, which does not focus on older adults with diabetes, we found that cognitive functioning is a predictor of functional disability [3,15-21]. Older adults with diabetes and low normal levels of cognition were approximately 20% more likely to die and 13% more likely to become disabled than those with higher levels of cognitive functioning over a 2-year period. This finding is alarming since accelerated declines in cognitive functioning have been consistently reported for older and middle-aged adults with diabetes [1-9].

The accelerated cognitive aging associated with diabetes suggests a more rapid aging of the central nervous system compared to older adults without diabetes [27]. The performance of people with diabetes on the TICS is equivalent to aging 4 years [6]. There are several plausible biological explanations of the relationship between diabetes and accelerated cognitive aging. Diabetes may interact with normative aging processes by additionally decreasing hippocampal volume resulting in cognitive decrements beginning in the 60s, manifesting in mainly memory-related changes [5]. Cerebro- and cardiovascular disease also might explain the association between diabetes and cognitive impairment because diabetes is a risk factor for stroke and hypertension both of which are predictors of dementia [6]. Abnormal levels of blood glucose at a cellular level could result in cerebro- and cardiovascular disease from failures to manage and monitor their diabetes properly [6].

Cognitive functioning has been identified as an important factor related to adherence by older adults to antihypertensive and arthritis treatment regimens [41] as well as remembering medical information [42]. Declining cognitive functioning in people with diabetes can have implications for adherence to treatment and to medication regimens, [9] the latter an IADL task. Sinclair and colleagues [2] reported that people who had diabetes and lower levels of cognitive functioning were less likely to be involved in monitoring their diabetes and in their treatment regimen, resulting in more hospitalizations, more ADL limitations, and the need for more personal assistance; thus, increased functional disability and mortality.

The relationship between cognitive functioning and adherence to treatment appears bidirectional. To minimize diabetes-related declines in cognitive functioning, older adults with diabetes should monitor their diabetes and adhere to treatment regimens [43]. It has been suggested that, in comparison with people with diabetes who do not adhere to their treatment regimen, cognitive decline is less in people adhering to either a monotherapy (use of sulfonylurea, metformin, or thiazolidinedione) or combination (sulfonylurea with another glucose-lowering agent, insulin, or metformin and insulin) [2,43], but other research does not support this finding with people who are using short-term glycemic control.[20]

Changes in performance on tasks of cognitive functioning may quickly and efficiently demonstrate changes in neuropsychological functioning and serve as a warning symptom for hypoglycemia [6]. That also can be an early indicator of future functional disability. The importance of cognition on functional disability was noted by observing the abilities of older adults that allow them to live independently, such as cognitive functioning, might be more important in predicting functional disability, institutionalization, and subsequently death than their medical diagnoses [2]. This is especially of concern for older adults with diabetes, considering the reported accelerated rates of cognitive decline in this group versus those without diabetes [1,44].

Our study demonstrates the potential of basic cognitive screening measures, which could have implications for health-care providers. Several brief measures to assess cognitive functioning are available that have respectable psychometric properties, such as the TICS, [27] Mini-Mental State Exam, Modified Mini-Mental State Exam, and the Short Portable Mental Status Questionnaire [45]. These instruments could allow patients who might be at the greatest risk of cognitive impairment, and subsequent death or functional disability to be identified and targeted for interventions.

Practitioners should keep in mind that depression has been shown to impair cognitive functioning, which is important because the rates of depression are higher in people with diabetes [8]. Bruce and colleagues [46] reported that in their sample of people with diabetes who were 70 years old and older, 14.2% were clinically depressed, 50.2% reported one or more depressive symptoms, and only 36% were free of depressive symptoms.

Various cognitive interventions have been successful at reversing age-related decrements in the cognitive components of inductive reasoning and spatial ability over 7 years, [42] improving the IADL task of managing medications, [47] and improving short-term memory performance in patients with Alzheimer's disease [48]. A challenge for planners and policy makers is to understand the heterogeneity of the trajectory of functional disability, as some persons steadily decline, resulting in their institutionalization or death, while others experience fluctuations and reversals of functional disability [12,36]. Understanding predictors of functional disability and how interventions relate to its incidence might provide some insight.

Our study has several limitations. First, as in other studies [19], functional disability outcomes were assessed using respondent-reported ADL and IADL measures instead of performance-based tasks. Although, performance-based measures might be a desired method of assessment, they may not reflect adaptations a person makes to successfully complete a task, but respondent-reported ADL measures can reduce the amount of missing data. Second, unfortunately, medication adherence and depression were not assessed on the LSOA II, which can potentially impair cognition. Despite these limitations, our prospective study contributes to the literature by emphasizing the importance of cognitive functioning in the development of functional disability and mortality in a representative population of older adults with diabetes who were free of cognitive impairment.

## Conclusion

Cognitive functioning among persons with diabetes is predictive of death and the development of functional disability over a 2-year interval. Older adults with diabetes and low normal levels of cognition, yet within normal ranges, were approximately 20% more likely to die and 13% more likely to become disabled than those with higher levels of cognitive functioning over a 2-year period. Health-care providers should consider using brief cognitive screening measures as indicators of mortality and disablement in their older patients with diabetes. This will allow at-risk older adults with diabetes to be targeted for interventions minimizing cognitive decline and functional disability, as well as targeting interventions and educational materials focusing on adherence to treatment and diabetes monitoring to appropriate individuals. These interventions potentially could minimize accelerated cognitive aging associated with diabetes; thus, delaying mortality and the development of functional disability.

## Competing interests

The author(s) declare that they have no competing interests.

## Authors' contributions

All authors contributed to the conception and design of the study, statistical analyses and interpretation of the data, revising the manuscript for critical content, and approved the final manuscript.

## Funding source

None

## Pre-publication history

The pre-publication history for this paper can be accessed here:


